# Intrapulmonary pharmacokinetics of SPR719 following oral administration of SPR720 to healthy volunteers

**DOI:** 10.1128/aac.01103-24

**Published:** 2024-10-01

**Authors:** Keith A. Rodvold, Mark H. Gotfried, Xilla T. Ussery, Shekman L. Wong, Kamal A. Hamed

**Affiliations:** 1 University of Illinois Chicago, Chicago, Illinois, USA; 2 Pulmonary Associates, PA, Phoenix, Arizona, USA; 3 Spero Therapeutics, Inc., Cambridge, Massachusetts, USA; St. George's, University of London, London, United Kingdom

**Keywords:** SPR720, alveolar macrophages, epithelial lining fluid, pharmacokinetics, pulmonary

## Abstract

SPR720 is a phosphate ester prodrug that is converted rapidly *in vivo* to SPR719, the active moiety, which exhibits potent *in vitro* activity against clinically relevant mycobacterial species including *Mycobacterium avium* complex (MAC) and *Mycobacterium abscessus*. SPR720 is in clinical development for the treatment of nontuberculous mycobacterial pulmonary disease (NTM-PD) due to MAC. This study evaluated the safety and the intrapulmonary pharmacokinetics of SPR719 in healthy volunteers. A total of 30 subjects received oral SPR720 1,000 mg once daily for 7 days followed by bronchoscopy and bronchoalveolar lavage, with blood samples collected for plasma pharmacokinetic assessments. Mean SPR719 area under the concentration-time curve from time 0 to 24 hours (AUC_0–24_) and maximum concentration (*C*_max_) for plasma, epithelial lining fluid (ELF), and alveolar macrophages (AM) were 52,418 ng·h/mL and 4,315 ng/mL, 59,880 ng·h/mL and 5,429 ng/mL, and 128,105 ng·h/mL and 13,033 ng/mL, respectively. The ratios of ELF to total plasma concentrations of SPR719 based on AUC_0–24_ and *C*_max_ were 1.14 and 1.26, and the ratios of AM to total plasma concentrations of SPR719 based on AUC_0–24_ and *C*_max_ were 2.44 and 3.02, respectively. When corrected for protein binding, the ratios of ELF to unbound plasma concentrations of SPR719 for AUC_0–24_ and *C*_max_ were 19.87 and 21.88, and the ratios of AM to unbound plasma concentrations of SPR719 for AUC_0–24_ and *C*_max_ were 42.50 and 52.53, respectively. No unexpected safety findings were observed. Results from this study of the intrapulmonary disposition of SPR719 support further investigation of SPR720 as a potential oral agent for the treatment of patients with NTM-PD.

This study is registered with Clinicaltrials.gov as NCT05955586.

## INTRODUCTION

Nontuberculous mycobacteria (NTM) represent a diverse group of organisms that cause chronic, progressive pulmonary disease primarily acquired from inhalation of mycobacteria in the environment, particularly in water and soil (1). The risk of NTM pulmonary disease (NTM-PD) is greater in those with underlying lung disease or impaired immunity and presents a challenge for diagnosis and treatment (2–4). *Mycobacterium avium* complex (MAC, which includes *M. avium* and *Mycobacterium intracellulare*) is the most common cause of NTM-PD, followed by *Mycobacterium abscessus, Mycobacterium xenopi*, *Mycobacterium fortuitum*, and *Mycobacterium kansasii* (5). Worldwide, the incidence and prevalence of NTM-PD are increasing (5). The increased prevalence of NTM-PD is accompanied by increased healthcare costs, especially among those not receiving guideline-directed therapy, and greater morbidity and mortality (6–8).

Amikacin liposome inhalation suspension, approved for treatment of refractory MAC pulmonary disease (MAC-PD), reduces hospitalization rates and healthcare resource use but is associated with a high rate of adverse events (9, 10). However, no oral antimicrobial agents are currently approved for the treatment of NTM-PD in the USA, Europe, or Japan, and consequently, recommendations from treatment guidelines are based on limited clinical data (11). Recommended treatment for NTM-PD varies by mycobacterial isolate and is guided by *in vitro* susceptibility testing. Treatment for MAC-PD typically involves a three-drug regimen including azithromycin and ethambutol for at least 12 months after sputum cultures convert from positive to negative (11, 12). Despite prolonged treatment with combination regimens, outcomes are poor and accompanied by intolerability, toxicity, high relapse rates, and increased mortality (6, 8, 13, 14). Thus, new treatment options for NTM-PD that provide improved safety and efficacy are needed (15).

SPR720 is being developed initially for the treatment of NTM-PD due to MAC (16). SPR720, an aminobenzimidazole, is an oral, chemically stable phosphate ester prodrug that is converted rapidly *in vivo* to SPR719, the active moiety. SPR719 targets in mycobacteria the ATPase subunits of DNA gyrase B, a mechanism that is distinct from that of fluoroquinolones, which inhibit DNA gyrase A (16). SPR719 exhibits potent *in vitro* activity against clinically relevant MAC species, *M. abscessus*, *M. fortuitum*, *M. kansasii*, and *Mycobacterium marinum*, and demonstrates no cross-resistance to other antibiotics used for treatment of NTM-PD (Table 1) (17–20). The *in vitro* activity of SPR719 is better than that of clarithromycin for MAC and other NTM species (18, 19). *In vivo* studies indicate that SPR719 is effective against mycobacterial species in murine models of infection and hollow fiber studies, and the efficacy of SPR719 is additive against MAC in combination with clarithromycin and ethambutol (20–23). At doses of 750–1,000 mg daily, SPR720 is predicted to achieve exposures associated with a bactericidal effect in 95% of patients (22). These results support further clinical evaluation of SPR720 for the treatment of NTM-PD.

**TABLE 1:** Activity of SPR719 against NTM clinical isolates from diverse specimen sources from patients in the USA and Japan between 2015 and 2018

		SPR719 MIC (µg/mL)		
NTM^*a*^ organism	Number of isolates	Range	MIC_50_	MIC_90_
*Mycobacterium avium* complex	105	0.002–4	1	2
*Mycobacterium abscessus*	53	0.12–8	2	4
*Mycobacterium fortuitum*	10	0.06–1	0.25	1
*Mycobacterium kansasii*	8	0.002–0.03	0.015	0.03
*Mycobacterium marinum*	9	0.12–1	0.5	0.5

^*a*^ NTM, nontuberculous mycobacteria.

Concentrations of antibiotics in pulmonary epithelial lining fluid (ELF) are important for determining antibiotic activity and optimal dosing in patients with pneumonia (24, 25). In particular, effective antimycobacterial therapy requires adequate uptake into alveolar macrophages (AM) where mycobacteria reside and proliferate (26). This study was designed to determine the concentrations of SPR719 in ELF and AM compartments of the lung to provide essential dose selection information for the development of SPR720 for the treatment of NTM-PD. The main objectives of this study were to evaluate the safety and intrapulmonary pharmacokinetics (PK), including ELF and AM concentrations of SPR719 compared to plasma concentrations of SPR719 in healthy adult subjects.

## RESULTS

The safety population comprised 33 healthy adult subjects, and the PK population included 30 subjects; three subjects discontinued the study early at physician discretion and were excluded from the PK analyses. Baseline characteristics for the PK population are shown in Table 2.

**TABLE 2:** Baseline characteristics of healthy adult subjects enrolled in the PK population^*a*^

Characteristic	Number (%) of subjects (*N* = 30)
Age, years^*b*^	43.5 ± 7.9
Age range, years	25–55
Male, n (%)	22 (73.3)
Race, n (%)	
Asian	1 (3.3)
Black or African American	4 (13.3)
White	21 (70.0)
Other	4 (13.3)
Hispanic or Latino, n (%)	14 (46.7)
Weight, kg^*b*^	83.8 ± 10.3
Body mass index (BMI), kg/m^2*b*^	27.9 ± 3.0
Forced expiratory volume at 1 second (FEV_1_), L^*b*^	3.6 ± 0.6
Forced expiratory volume at 1 second (FEV_1_), % predicted^*b*^	98.9 ± 10.2

^*a*^ Subjects who received at least one dose of SPR720 with at least one evaluable plasma concentration and a respiratory PK sample. Three subjects discontinued the study and were excluded from the PK population. One subject was a 47-year-old male, Black or African American, not Hispanic or Latino, weighed 87.4 kg, and had a BMI of 26.9 kg/m^2^; the second was a 51-year-old male, with race listed as “other,” Hispanic or Latino, weighed 74.9 kg, and had a BMI of 26.6 kg/m^2^; and the third was a 41-year-old female, White, Hispanic or Latino, weighed 64.8 kg, and had a BMI of 24.7 kg/m^2^.

^*b*^ Mean ± SD.

### Pharmacokinetics

**Fig 1:**
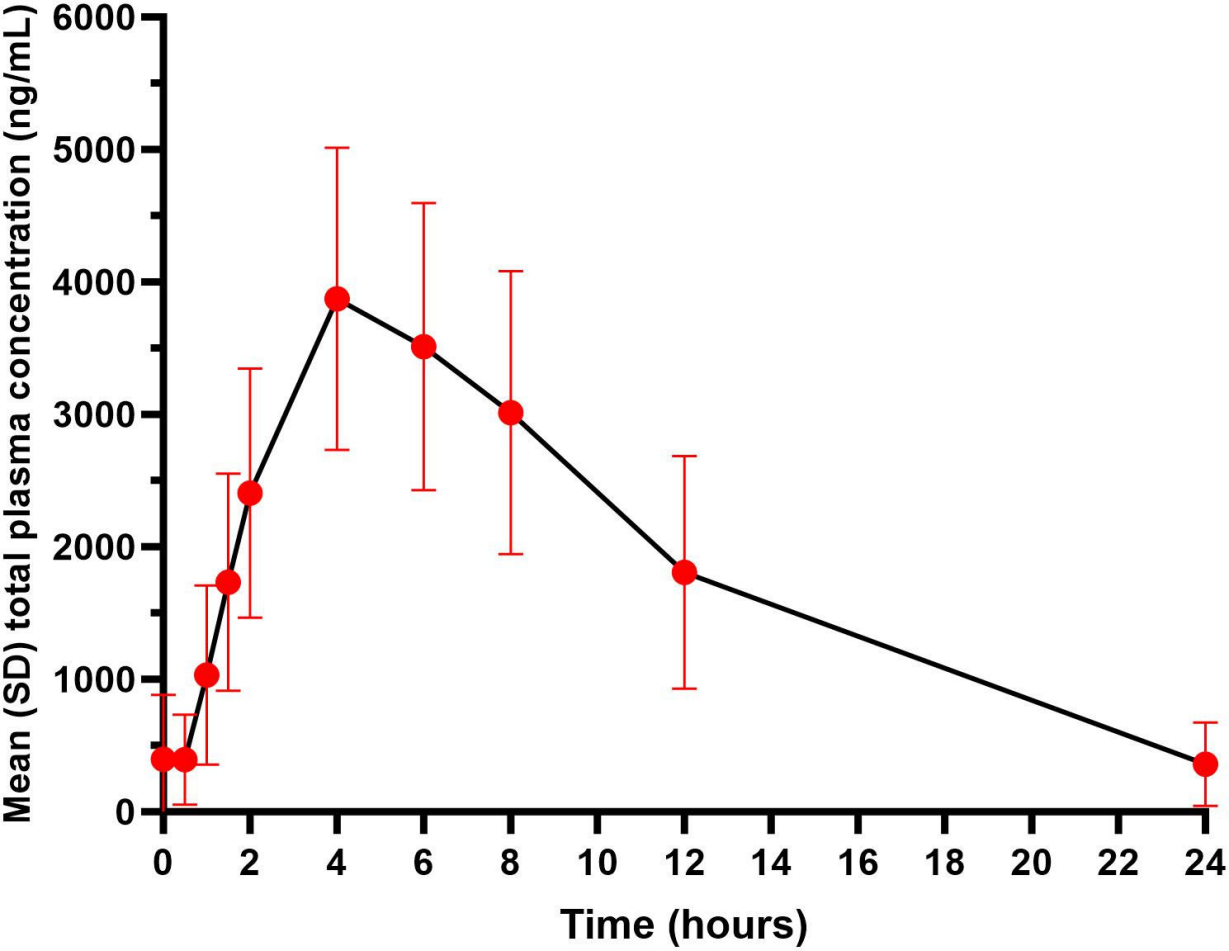
Mean (±SD) total plasma SPR719 concentrations after 7 days of SPR720 1,000 mg daily (PK population). PK population included all the subjects who received at least one dose of SPR720 with at least one evaluable plasma concentration and a respiratory PK sample.

There was no meaningful detectable plasma concentration of SPR720. Mean plasma concentrations of SPR719 reached a peak at approximately 4 hours and then declined over the remaining 24 hours (Fig. 1). After 7 days of dosing, SPR719 mean half-life (*t*_1/2_) was approximately 5 hours, time to maximum plasma concentration (*T*_max_) was 4 hours, mean (standard deviation [SD]) maximum concentration (*C*_max_) was 4,187 (1,059) ng/mL, and area under the concentration-time curve (AUC) from time 0 to 24 hours (AUC_0–24_) was 42,295 ng·h/mL (Table 3).

**TABLE 3:** Plasma PK parameters for SPR719 after 7 days of SPR720 1,000 mg daily (PK population)^*a*^

	*C*_max_^*b*^ (ng/mL)	*T*_max_^*c*^ (hours)	AUC_0–last_^*d*^ (ng·h/mL)	AUC_0–24_^*e*^ (ng·h/mL)	*t*_1/2_^*f*^ (hours)	*C*_24_^*g*^ (ng/mL)
All subjects (*N* = 30)	4,187 ± 1059	4.0 (2.0–8.0)	42,295 ± 14,982	42,295 ± 14,982	4.97 ± 1.54	359 ± 314
Cohort 1: 1.5 hours post-dose (*n* = 6)	4,288 ± 1061	4.0 (2.0–6.0)	43,974 ± 22,072	43,974 ± 22,079	5.91 ± 1.77	508 ± 452
Cohort 2: 4 hours post-dose (*n* = 6)	4,440 ± 1613	4.0 (4.0–6.0)	40,253 ± 18,019	40,253 ± 18,019	5.14 ± 2.01	344 ± 381
Cohort 3: 8 hours post-dose (*n* = 6)	4,305 ± 829	4.0 (4.0–6.0)	44,672 ± 14,447	44,672 ± 14.447	4.73 ± 1.37	351 ± 298
Cohort 4: 12 hours post-dose (*n* = 6)	4,188 ± 893	6.0 (4.0–8.0)	44,707 ± 9,990	44,707 ± 9,990	4.70 ± 1.38	350 ± 246
Cohort 5: 24 hours post-dose (*n* = 6)	3,713 ± 958	4.0 (4.0–6.0)	37,867 ± 11,652	37,867 ± 11,652	4.36 ± 1.08	241 ± 173

^*a*^ PK population included all the subjects who received at least one dose of SPR720 with at least one evaluable plasma concentration and a respiratory PK sample. Data are arithmetic mean ± SD except for *T*_max_ (median [range]).

^*b*^ *C*_max_, maximum plasma concentration.

^*c*^ *T*_max_, time to *C*_max_.

^*d*^ AUC_0–last_, area under the plasma concentration-time curve from time 0 to last assessment.

^*e*^ AUC_0–24_, area under the plasma concentration-time curve from time 0 to 24 hours.

^*f*^ *t*_1/2_, half-life.

^*g*^ *C*_24_, plasma concentration at 24 hours.

**Fig 2:**
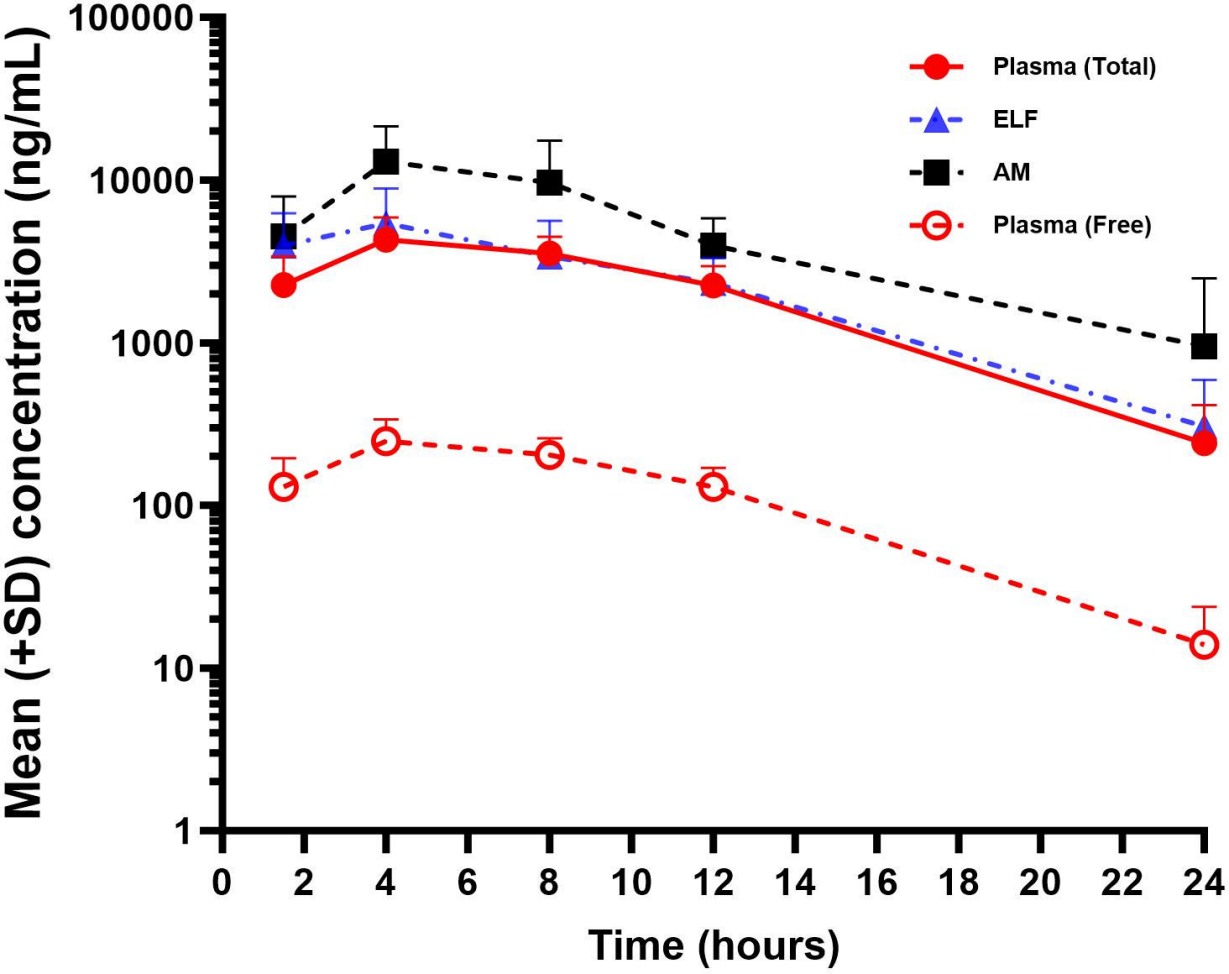
Mean (+SD) SPR719 concentrations in plasma (total and unbound), ELF, and AM after 7 days of SPR720 1,000 mg daily. AM, alveolar macrophages; ELF, epithelial lining fluid.

Simultaneous concentrations of SPR719 measured in plasma, ELF, and AM showed that mean (SD) concentrations of SPR719 were greater in ELF and AM than in plasma (Table 4). The corresponding mean SPR719 AUC_0–24_ and *C*_max_ for plasma were 52,418 ng·h/mL and 4,315 ng/mL, respectively, for ELF were 59,880 ng·h/mL and 5,429 ng/mL, respectively, and for AM were 128,105 ng·h/mL and 13,033 ng/mL, respectively. Across the 24-hour sampling period, mean (SD) SPR719 ELF and AM concentrations were substantially greater than unbound plasma SPR719 concentrations (Fig. 2).

**TABLE 4:** SPR719 concentrations in plasma, ELF, and AM at assigned BAL sampling times (PK population)^*a*^

	Arithmetic mean ± SD		
BAL^*b*^ sampling time post-dose (hours)	Plasma (total) (ng/mL)	ELF^*c*^ (ng/mL)	AM^*d*^ (ng/mL)
Cohort 1: 1.5 hours (*n* = 6)	2,261 ± 1,134	3,970 ± 2,306	4,508 ± 3,422
Cohort 2: 4 hours (*n* = 6)	4,315 ± 1,577	5,429 ± 3,463	13,033 ± 8,412
Cohort 3: 8 hours (*n* = 6)	3,545 ± 950	3,398 ± 2,209	9,637 ± 7,822
Cohort 4: 12 hours (*n* = 6)	2,258 ± 696	2,330 ± 986	3,970 ± 1,841
Cohort 5: 24 hours (*n* = 6)	241 ± 173	306 ± 287	952 ± 1,534

^*a*^ PK population included all the subjects who received at least one dose of SPR720 with at least one evaluable plasma concentration and a respiratory PK sample.

^*b*^ BAL, bronchoalveolar lavage.

^*c*^ ELF, epithelial lining fluid.

^*d*^ AM, alveolar macrophages.

Mean ratios of ELF and AM to total plasma concentrations for SPR719 at each sampling time during the 24-hour period ranged from 0.99 to 1.66 and 1.71 to 3.54, respectively. The ratios of ELF to total plasma SPR719 concentrations based on AUC_0–24_ and *C*_max_ values were 1.14 and 1.26, respectively. When corrected for protein binding (94.25% protein-bound fraction and 5.75% unbound fraction with standard equilibrium dialysis), the ratios of ELF to unbound plasma SPR719 concentrations based on AUC_0–24_ and *C*_max_ values were 19.87 and 21.88, respectively. The ratios of AM to total plasma SPR719 concentrations based on AUC_0–24_ and *C*_max_ values were 2.44 and 3.02, respectively. When corrected for protein binding, the ratios of AM to unbound plasma SPR719 concentrations based on AUC_0–24_ and *C*_max_ values were 42.50 and 52.53, respectively (Table 5).

**TABLE 5:** Ratios of ELF and AM AUC_0–24_ and *C*_max_ to total and unbound plasma AUC_0–24_ and *C*_max_ of SPR719 (PK population)^*a*^

	ELF^*b*^ to plasma AUC_0–24_^*c*^	ELF to plasma *C*_max_^*d*^	AM^*e*^ to plasma AUC_0–24_	AM to plasma *C*_max_
Total plasma	1.14	1.26	2.44	3.02
Unbound plasma	19.87	21.88	42.50	52.53

^*a*^ PK population included all the subjects who received at least one dose of SPR720 with at least one evaluable plasma concentration and a respiratory PK sample.

^*b*^ ELF, epithelial lining fluid.

^*c*^ AUC_0-24_, area under the concentration-time curve from time 0 to 24 hours.

^*d*^ *C*_max_, maximum concentration.

^*e*^ AM, alveolar macrophages.

### Safety

At least one treatment-emergent adverse event (TEAE) was reported by 19 (57.6%) subjects (Table 6). The events were mild in 17 (51.5%) subjects and moderate in two (6.1%) subjects. Fifteen (45.5%) subjects had drug-related TEAEs that were mostly of mild severity. Three (9.1%) subjects discontinued study drug early at physician discretion based on minor blood chemistry findings. Two subjects with normal serum creatinine levels at baseline had elevated creatinine levels (1.76 and 1.49 mg/dL; Common Terminology Criteria for Adverse Events [CTCAE] grade 1) at Day 6, which returned to normal levels at Day 8 and Day 15 visits. One subject with a serum bilirubin level of 1.4 mg/dL at baseline had a bilirubin level of 2.2 mg/dL at Day 4 (CTCAE grade 2) that returned to normal levels at Day 8 and Day 15 visits. No serious adverse events were reported. No clinically significant changes in vital signs, physical examination, or electrocardiogram (ECG) were noted. At the Day 8 and Day 15 visits, all QT interval values using Fridericia correction (QTcF) were ≤450 ms, and no subjects experienced an increase in QTcF >30 ms.

**TABLE 6:** Incidence of TEAEs (safety population)^*a*^

	Number (%) of subjects *N* = 33
Any TEAE^*b*^	19 (57.6)
Any drug-related TEAE	15 (45.5)
Any TEAE leading to study drug discontinuation	3 (9.1)
Individual TEAEs	
Diarrhea	13 (39.4)
Nausea	5 (15.2)
Abdominal distension	2 (6.1)
Headache	3 (9.1)
Blood creatinine increased	2 (6.1)
Neutropenia	1 (3.0)
Hyperbilirubinemia	1 (3.0)
Post-procedural fever	1 (3.0)

^*a*^ Safety population included all the subjects who received at least one dose of SPR720. Subjects may have reported more than one TEAE.

^*b*^ TEAE, treatment-emergent adverse event.

## DISCUSSION

For lower respiratory tract infections, studies using bronchoscopy and bronchoalveolar lavage (BAL) are valuable to assess the intrapulmonary penetration of antibiotics into ELF and AM as confirmation that antibiotic levels reach the site of infection. In this study, the intrapulmonary disposition of SPR719 was investigated in healthy subjects, and concentrations of SPR719 in ELF and AM were found to be greater than total plasma concentrations. These results suggested that SPR719 had significant lung uptake and enhanced ELF and AM concentrations because unbound plasma concentrations predominantly influence penetration into lung compartments.

In a previous single- and multiple-ascending dose study, one cohort of subjects received oral SPR720 1,000 mg daily for 7 days (27). In the PK analysis, *T*_max_ was 4.0 hours, *t*_1/2_ was 2.9 hours, *C*_max_ was 5,110 ng/mL, and AUC_0–24_ was 30,600 ng·h/mL. In the multiple-ascending dose phase, 60% of subjects experienced at least one TEAE, most commonly diarrhea, nausea, abdominal pain/discomfort, or headache. Thus, the PK and safety findings from the current study were consistent with the PK and safety results reported from this previous study. Data from both studies will be used in a population PK model, which, together with nonclinical pharmacokinetic-pharmacodynamic (PK-PD) targets for efficacy, will support PK-PD target attainment and dose selection.

Intrapulmonary PK of a wide range of antibiotics have been evaluated in healthy subjects and critically ill patients where ELF-to-plasma ratios were most often 0.5 or less with beta-lactams but greater than 1.0 with ketolides, fluoroquinolones, tetracyclines, and tedizolid (25). However, few studies have evaluated the intrapulmonary PK of drugs for treating NTM-PD despite the importance of establishing adequate pulmonary disposition to support efficacy. A previous study evaluated the intrapulmonary PK of an intravenous (IV) formulation of epetraborole in healthy volunteers (28). Subjects received a 1,500-mg IV dose for 5 days, and BAL was conducted on Day 1 and Day 3. At Day 3, epetraborole *C*_max_ was 24.0 µg/mL, and AUC_0-12_ was 72.9 µg·h/mL. The ELF-to-plasma ratios were 0.534 and 0.597 for AUC_0-12_ and *C*_max_, respectively, and the AM-to-plasma ratios were 4.98 and 5.47 for AUC_0-12_ and *C*_max_, respectively. Epetraborole concentrations in ELF and AM were highest at the 2-hour sampling time but decreased rapidly at 6 and 12 hours. As compared with epetraborole, SPR719 had greater ELF-to-plasma ratio, and when correcting for protein binding for both SPR719 and epetraborole, SPR719 also had greater AM-to-plasma ratio.

Limitations of this study include the assumption that there was no intrapulmonary protein binding of SPR719 when ELF and AM penetration ratios were calculated. Furthermore, plasma protein binding of SPR719 was determined in a previous study as noted in the Materials and Methods section.

Current treatment recommendations for NTM-PD include combinations of drugs that often require complicated dosing regimens and carry the potential for clinically significant drug–drug interactions, increased adverse events, and poor patient adherence (1, 2, 29–31). Thus, new treatment options are needed that offer the potential for improved safety and clinical outcomes (16). Results from this study of the intrapulmonary disposition of SPR719 support further considerations of SPR720 as a potential oral agent for the treatment of patients with NTM-PD caused by MAC.

## MATERIALS AND METHODS

### Study design

This was a Phase 1, single-center, open-label study in healthy adult male and female volunteers. Subjects received a 1,000-mg dose (four 250-mg capsules) of SPR720 administered once daily for 7 days. A screening evaluation to determine eligibility for enrollment was performed within 21 days of initial dosing. Subjects who satisfied inclusion and exclusion criteria visited the study site on Day −1 (1 day prior to start of dosing) for study assessments, and on Days 1 to 5 to receive study treatment and complete drug and alcohol screening and safety assessments. Subjects were confined to the study unit from Day 6 through Day 8 for bronchoscopy and study assessments. Subjects returned for a follow-up visit on Day 15 (±3 days) after discharge from the study site. The maximum duration of participation for each subject was up to 39 days.

### Study population

Eligible subjects were adults aged 18 to 55 years, with a body mass index (BMI) ≥18.0 and ≤32 kg/m^2^ and a body weight between 55.0 and 100.0 kg, and had been nonsmokers for at least 30 days prior to screening. Subjects were medically healthy without clinically significant abnormalities based on a screening medical history, physical examination, vital signs, 12-lead ECG, and clinical laboratory tests. Subjects had a forced expiratory volume in 1 second (FEV_1_) of at least 80% of predicted at screening; abstained from alcohol, caffeine, xanthine-containing beverages or food for 48 hours prior to and during the study; and agreed to use an effective method of contraception during the study.

At screening, subjects were excluded for a history of any significant medical condition; history of known or suspected *Clostridioides difficile* infection; positive urine drug, alcohol, or cotinine test; or positive test for human immunodeficiency virus, hepatitis B surface antigen, or hepatitis C antibody. Subjects with any clinically significant laboratory abnormalities or an ECG finding of a QTcF interval duration ≥450 ms for males and ≥470 ms for females were also excluded.

### Blood sample collection

Blood samples to measure plasma SPR720 and SPR719 concentrations were collected within 15 minutes before dosing on Day 6**,** within 15 minutes pre-dose (0 hour), and at 0.5, 1, 1.5, 2, 4, 6, 8, and 12 hours after the oral dose on Day 7. A final blood sample was collected on Day 8, 24 hours after the last dose taken on Day 7.

### Bronchoscopy and BAL

Each subject underwent one standardized bronchoscopy and BAL on Day 7. Subjects were assigned to one of five bronchoscopy sampling times at 1.5, 4, 8, 12, or 24 (±10 minutes) hours after the seventh dose of SPR720. A blood sample to determine plasma concentrations of SPR719 and urea was obtained during the bronchoscopy procedure at each BAL sampling time (approximating the time of collection of a second aspirate of BAL). Detailed descriptions of the bronchoscopy and BAL procedures, and the handling, processing, and storage of samples were described previously (32–34).

### Determination of plasma concentrations of SPR719

Concentrations of SPR719 in plasma were determined by a validated liquid chromatography with tandem mass spectrometry (LC-MS/MS) assay performed at QPS, LLC (Newark, DE, USA). The assay range was 10 to 10,000 ng/mL. Intra-day precision (% coefficient of variation [%CV]) ranged from 1.1% to 6.9%, and accuracy (% relative error [%RE]) ranged from –2.3% to 12.7%. Inter-day precision (%CV) ranged from 4.5% to 8.0%, and accuracy (%RE) ranged from 1.8% to 4.8%.

### Determination of BAL fluid and cell pellet concentrations of SPR719

The concentration of SPR719 in BAL and cell pellets was determined by Keystone Bioanalytical, Inc. (North Wales, PA, USA) using a validated LC-MS/MS method. ELF concentrations were calculated by the urea dilution method (35, 36). AM concentrations were determined from cell pellet drug concentrations, cell count in BAL fluid, and macrophage cell volume (36). The calibration curves for SPR719 were linear over a range from 0.25 to 100 ng/mL. The inter-assay precision (%CV) and accuracy (%RE) for SPR719 were 2.85% to 8.64% and –10.18% to 3.50%, respectively. The intra-assay precision (%CV) and accuracy (%RE) for SPR719 were 0.75% to 11.55% and –10.87% to 1.12%, respectively.

### Determination of urea concentration

The concentration of urea in plasma and BAL fluid supernatants was determined using a validated LC/MS/MS method from Keystone Bioanalytical (North Wales, PA, USA). The calibration curve for the urea assay in plasma was linear over the range from 100 to 3,000 µg/mL. The inter-assay precision (%CV) and accuracy (%RE) for urea were 1.98% to 3.37% and –8.17% to 4.13%, respectively. The intra-assay precision (%CV) and accuracy (%RE) for urea were 0.56% to 5.60% and –10.21% to 8.54%, respectively. The calibration range of the urea assay for BAL fluid was linear over the range of 0.2 to 10 µg/mL. The inter-assay precision (%CV) and accuracy (%RE) for urea were 6.40% to 9.77% and –3.22% to 1.47%, respectively. The intra-assay precision (%CV) and accuracy (%RE) for urea were 0.96% to 12.08% and –11.30% to 9.15%, respectively.

### Pharmacokinetic analysis

Pharmacokinetic analysis of SPR719 plasma concentrations was determined with noncompartmental PK analysis using Phoenix WinNonlin (version 8.3, Certara USA, Inc., Princeton, NJ, USA). The PK parameters were *C*_max_, *T*_max_, AUC_0–24_, AUC from time 0 to last assessment (AUC_0–last_), and plasma concentration at 24 hours (*C*_24_).

### Antibiotic concentrations for ELF and AM

Concentrations of SPR719 were summarized separately for plasma, ELF, and AM by nominal time point using descriptive statistics. Mean concentrations of SPR719 in plasma and BAL fluid (ELF and AM) obtained at the different sampling times were used to estimate the AUC_0–24_ for SPR719 in plasma, ELF, and AM. The concentration at the final sampling time (24 hours) also served as the time zero value for determining the AUC_0–24_ value of each matrix using Phoenix WinNonlin (version 8.3, Certara USA, Inc., Princeton, NJ, USA). The ratios of AUC_0–24_ for ELF or AM to plasma (total and unbound) were calculated. Plasma protein binding analyses were not conducted as part of this study. The value used for the unbound fraction of SPR719 in plasma was 5.75% (standard equilibrium dialysis from 0.5 to 10 µM) (Spero Therapeutics, Inc., data on file). Individual plasma concentration-time profile versus time and arithmetic mean concentration (±SD) versus time plots were presented for the concentration-time data.

Volume and drug concentrations for ELF and AM were calculated from BAL fluid supernatants and cell pellets from pooled aspirates (36). The concentration of SPR719 in ELF (*C*_ELF_) was determined as follows: *C*_ELF_ = concentration in BAL × BAL volume / ELF volume). ELF volume was derived using ELF volume = BAL volume × urea_BAL_ / urea_plasma_, where urea_BAL_ was the concentration of urea in BAL fluid and urea_plasma_ was the concentration of urea in plasma. The *C*_AM_ of SPR719 was determined using *C*_AM_ = *C*_pellet_ / V_AM_, where *C*_pellet_ was the mass of SPR719 measured in the cell suspension and V_AM_ was the volume of alveolar cells in the 1-mL cell suspension. A mean macrophage cell volume of 2.42 µL/10^6^ cells was used for V_AM_ (37, 38).

### Safety assessments

Subjects were continuously monitored during bronchoscopy. Vital signs (blood pressure, heart rate, respiratory rate, and temperature) were recorded prior to the scheduled bronchoscopy time and at 15 minutes and 1 hour following the end of bronchoscopy. Subjects were observed for 2 to 3 hours post-bronchoscopy for safety evaluations. Continuous pulse oximetry was monitored from when the subject was prepped for bronchoscopy until the post-procedure oxygen saturation was at least 93% on room air. Pulse oximetry was extended if a clinically significant increase in heart rate or cardiac rhythm abnormalities were detected. Other safety assessments included physical examinations, clinical laboratory monitoring (hematology, blood chemistry, urinalysis), 12-lead ECG, and adverse events.

### Statistical analysis

The study was primarily descriptive, comparing the steady-state concentrations of SPR719 in plasma, ELF, and AM at selected time intervals. Thus, no formal statistical hypothesis testing was performed. The sample size was based on the need to obtain adequate safety, tolerability, and PK data to achieve the objectives of the study while exposing a minimum number of subjects to study medication and procedures. A sample size of six subjects per cohort at each BAL time point (i.e., a total of 30 subjects) was considered sufficient. Phoenix WinNonlin (version 8.3, Certara USA, Inc., Princeton, NJ, USA) was used to analyze the data and create summary tables, subject data listings, and graphical representations.
